# Association between long-term adherence to class-I recommended medications and risk for potentially preventable heart failure hospitalizations among younger adults

**DOI:** 10.1371/journal.pone.0222868

**Published:** 2019-09-23

**Authors:** Tiffany E. Chang, Soyoun Park, Quanhe Yang, Fleetwood Loustalot, Javed Butler, Matthew D. Ritchey

**Affiliations:** 1 Division for Heart Disease and Stroke Prevention, National Center for Chronic Disease Prevention and Health Promotion, Centers for Disease Control and Prevention, Atlanta, GA, United States of America; 2 IHRC, Inc., Atlanta, GA, United States of America; 3 University of Mississippi Medical Center, Jackson, MS, United States of America; Ball State University, UNITED STATES

## Abstract

**Background:**

Five guideline-recommended medication categories are available to treat patients who have heart failure (HF) with reduced ejection fraction. However, adherence to these medications is often suboptimal, which places patients at increased risk for poor health outcomes, including hospitalization. We aimed to examine the association between adherence to these medications and potentially preventable HF hospitalizations among younger insured adults with newly diagnosed HF.

**Methods and results:**

Using the 2008–2012 IBM MarketScan Commercial database, we followed 26,439 individuals aged 18–64 years with newly diagnosed HF and calculated their adherence (using the proportion of days covered (PDC) algorithm) to the five guideline-recommended medication categories: angiotensin-converting enzyme inhibitors/angiotensin-receptor blockers; beta blockers; aldosterone receptor antagonists; hydralazine; and isosorbide dinitrate. We determined the association between PDC and long-term preventable HF hospitalizations (observation years 3–5) as defined by the United States (U.S.) Agency for Healthcare Research and Quality. Overall, 49.0% of enrollees had good adherence (PDC≥80%), which was more common among enrollees who were older, male, residing in higher income counties, initially diagnosed with HF in an outpatient setting, and who filled prescriptions for fewer medication categories assessed. Adherence differed by medication category and was lowest for isosorbide dinitrate (PDC = 60.7%). In total, 7.6% of enrollees had preventable HF hospitalizations. Good adherers, compared to poor adherers (PDC<40%), were 15% less likely to have a preventable hospitalization (HR 0.85, 95% confidence interval, 0.75–0.96).

**Conclusion:**

We found that approximately half of insured U.S. adults aged 18–64 years with newly diagnosed HF had good adherence to their HF medications. Patients with good adherence, compared to those with poor adherence, were less likely to have a potentially preventable HF hospitalization 3–5 years after their initial diagnosis. Because HF is a chronic condition that requires long-term management, future studies may want to assess the effectiveness of interventions in sustaining adherence.

## Introduction

Nearly 6.2 million adults age 20 years or older have heart failure (HF) in the United States (U.S.) [1]. These individuals have a high risk for morbidity and mortality and place a considerable burden on the healthcare system. The total direct and indirect cost of HF in the U.S. is estimated to be over $30 billion annually, with the majority attributed to inpatient hospital care [2]. HF is considered to be an ambulatory care sensitive condition, which are conditions for which provision of good outpatient care can minimize the occurrence of acute hospitalizations [3]. Yet, around one million HF hospitalizations that are considered potentially preventable occur among U.S. adults age 18 or older each year, and one-quarter of these occur among those aged 18–64 years (approximately 250,000 hospitalizations) [4].

To address the sizable burden of preventable HF hospitalizations, optimizing care in the outpatient setting to improve HF management and prevent acute events has been a national priority [5, 6]. The American College of Cardiology and American Heart Association’s (ACC/AHA) 2013 national HF management guideline lists five specific class I-recommended medication categories for the management of HF with reduced ejection fraction (HFrEF) [7]. However, adherence to these medications appears to be important for the long-term management of HF regardless of ejection fraction status [8]. Prior studies that have examined a subset of guideline-recommended medication categories have found that adherence is associated with improved outcomes, such as decreased risk for hospitalization [9–11], reduced healthcare costs [12, 13], and improved survival [9, 11, 14].

Patients with HF require lifelong management, including optimization of medication therapy, to maximize their cardiac function and minimize disease progression. However, most studies have examined medication adherence and its association with outcomes among persons with HF only in the short-term (less than two years) [10, 15–17]. Furthermore, we are unaware of any large studies examining these associations exclusively among younger adults with HF, among whom the long-term implications of medication adherence may even be more important. Accordingly, this study assesses adherence to the five ACC/AHA guideline-recommended medication categories, individually and collectively, among individuals aged 18–64 years with newly diagnosed HF and its association with long-term (3–5 year) risk for having a preventable HF hospitalization.

## Methods

### Study population

**Fig 1:**
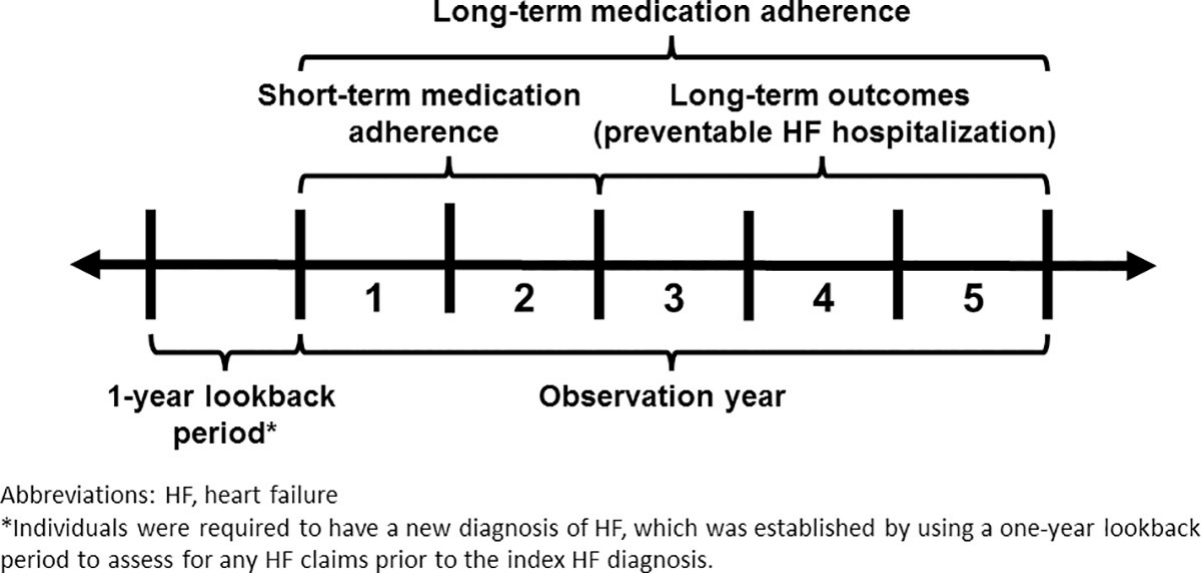
Definitions of the look-back period, short-term adherence, long-term adherence, and long-term preventable HF hospitalizations by observation years.

This study used the 2008–2012 IBM MarketScan Commercial database, which is a national convenience sample that captures medical and prescription claims from more than 300 employers and 25 commercial health plans [18]. Enrollees (i.e., individuals with health plans included in MarketScan) were eligible for inclusion in the study cohort if, during January 1, 2008-December 31, 2010, they were aged 18–64 years and had at least one inpatient or outpatient claim for HF (ICD-9-CM codes 428.x, 402.01, 402.11, 402.91, 404.01, 404.11, and 404.91). Enrollees with HF diagnoses related to end-stage renal disease (ICD-9-CM code 404.x3) were excluded. The first qualifying HF diagnosis was referred to as the index diagnosis. Enrollees were also required to have at least two medication fills in the same category for at least one of the included HF-related medication categories that occurred after the index diagnosis and before having a preventable HF hospitalization [19], as well as a qualifying prescription fill measurement period of at least 90 days. The first of the qualifying prescription fills was referred to as the index fill. Enrollees were also required to have continuous coverage within a health plan included in the MarketScan database for at least 90% of the calendar days each year starting 365 days prior to the index HF diagnosis date and then yearly thereafter until they had a preventable HF hospitalization, they died while hospitalized (enrollees who died outside of the hospital were unable to be identified), or the study ended (December 31, 2012). Finally, enrollees were required to have a new HF diagnosis, which was established by using a one-year lookback period from the date of the index HF diagnosis to assess for any prior HF claims (Fig 1). Enrollees with a previous HF claim were excluded.

**Fig 2:**
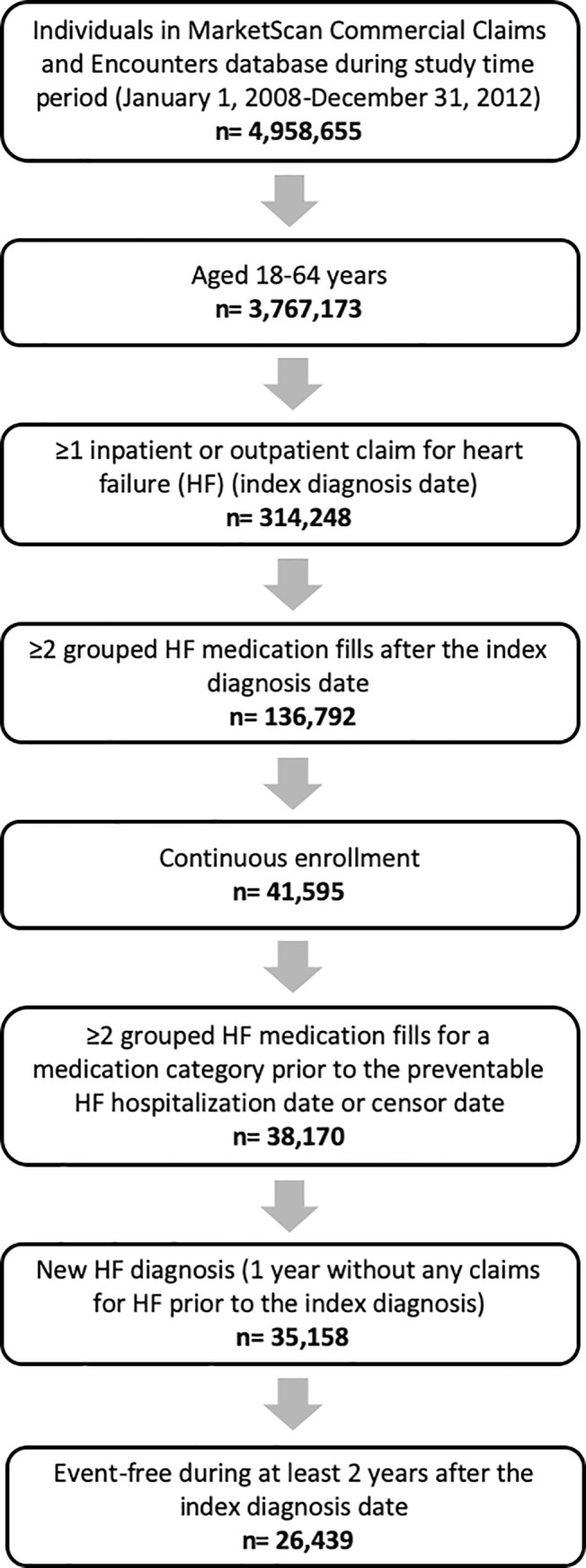
Enrollee cohort selection based on inclusion criteria during enrollment (January 1, 2008- December 31, 2010), MarketScan 2008–2012.

MarketScan data from 2008–2010 included a total of 4,958,655 unique enrollees. After applying the inclusion criteria, 33,158 individuals remained in the study (Fig 2). To examine the association between medication adherence and long-term risk of preventable HF hospitalizations, an additional 6,719 enrollees were excluded due to death or having a preventable HF hospitalization occur within two years of their index diagnosis (i.e., during the short-term period) (4,587 excluded during the year one; 2,132 during year two). The final study cohort included 26,439 enrollees.

### Heart failure medication categories and adherence

Adherence was assessed for the five class-I ACC/AHA guideline-recommended prescription medication categories for HFrEF: (1) angiotensin-converting enzyme inhibitors (ACEIs) or angiotensin-receptor blockers (ARBs); (2) beta blockers (specifically carvedilol, bisoprolol, sustained-release metoprolol succinate); (3) aldosterone receptor antagonists (spironolactone, eplerenone); (4) hydralazine; and (5) isosorbide dinitrate [7]. To be maximally inclusive, medication preparations within these categories were identified using classification schema from three sources: MarketScan Red Book, IMS Health Uniform System of Classification codes [20], and the U.S. Food and Drug Administration’s National Drug Codes [21].

Medication adherence was calculated for each medication category using the proportion of days covered (PDC) algorithm. This method is endorsed and utilized by multiple organizations [22–24]. For each enrollee, PDC was calculated as the proportion of days the individual had prescription fills for a particular medication category starting from the index fill date until the study end date, date of a preventable HF hospitalization or date of death while hospitalized. Inpatient hospitalization days for any cause were excluded from the PDC calculation. Based on prior studies, good adherence was defined as PDC ≥80% [25, 26], and then the PDC <80% group was further categorized as having moderate (PDC 40-<80%) or poor (PDC <40%) adherence [27, 28] to assess for associations between these levels of adherence and hospitalizations. For enrollees who filled prescriptions for more than one included medication category, an unweighted simple mean of the PDCs for each category was calculated. These enrollees could be taking the multiple medication categories concurrently or at different times (potentially switching medication categories). Additionally, for enrollees who were prescribed more than one medication category at any time during the study, the top three combinations of medication categories were described.

We defined PDC during the full analysis period (observation years 1–5) as “long-term adherence”, which reflects adherence during the study’s full observation period (Fig 1). Additionally, we incorporated a measure of “short-term adherence”, which was restricted to adherence during observation years 1–2.

### Preventable heart failure hospitalizations

Preventable HF hospitalizations were defined using the U.S. Agency for Healthcare Research and Quality’s Prevention Quality Indicator #8, which is for HF admission rates. Briefly, all non-maternal/non-neonatal inpatient hospital discharges for individuals aged 18 or older with a principal diagnosis for HF were classified as being potentially preventable; these excluded transfers from other institutions and hospitalizations with certain cardiac procedures (e.g., repair/replacement of pacemakers and heart valves) [19]. In effect, this indicator aims to exclude planned hospitalizations and only identify acute hospitalizations that are potentially preventable if high-quality outpatient care had been provided.

### Baseline covariates

The following variables were included as baseline covariates: age group at study enrollment (18–34, 35–44, 45–54, 55–64 years), sex, employment status (full-time, part-time, disabled, retired, other), U.S. Census Bureau geographic regions (northeast, north central, south, west), preventable HF hospitalization, number of HF medication categories prescribed, HF diagnosis setting, and a modified version of the Charlson Comorbidity Index (CCI) [29]. Additionally, data from the U.S. Census Bureau’s Area Resource File of the 2006–2010 five-year county-level median household income were categorized into tertiles (low: <$38,952; medium: $38,952-<46,334; high: ≥$46,334) and linked to enrollee’s county of residence [30]. The diagnosis setting was determined by using the location where the index diagnosis claim was submitted (inpatient or outpatient file within the MarketScan data). If an enrollee’s index HF diagnosis occurred in both the inpatient and outpatient file on the same date, it was classified as an inpatient diagnosis.

### Statistical analysis

In descriptive analyses we determined the proportion of individuals in each PDC category for the aforementioned covariates and each medication category. The Chi-square test was used to determine statistical differences across PDC categories. Analysis years were defined as “observation years”, with observation year one starting on the date of each enrollee’s index diagnosis. At most, enrollees could be followed up until the end of observation year five. To assess for changes in enrollees’ PDC classification over time, a descriptive analysis was conducted to describe how enrollees’ PDC values were characterized in the short-term (observation years 1–2) versus the long-term (observation years 1–5; Fig 1).

To examine the association between adherence and long-term risk of preventable HF hospitalizations (events during observation years 3–5), Cox proportional hazard models were constructed to estimate hazard ratios (HR) and 95% confidence intervals (CI), with PDC <40% used as the referent group. Two separate models were used to measure long-term risk for preventable HF hospitalization models: a primary long-term adherence model, and as a sensitivity analysis, a secondary short-term adherence model. The proportional hazards assumption was tested in Cox models by creating time-dependent interaction terms and testing if the outcome effect differed by any baseline covariates [31]. A covariate-adjusted cumulative incidence curve was constructed to compare long-term incidence of preventable HF hospitalizations among enrollees by long-term adherence category (poor, moderate, or good adherence).

In secondary analyses, the association between long-term adherence and preventable HF hospitalizations was examined for the three most frequently used combinations of medication categories (from concurrent use or switching). *P*<0.05 was used to indicate statistical significance. All analyses were conducted using SAS version 9.3 (Cary, NC).

## Results

### Baseline characteristics and medication use

The full study cohort included 26,439 enrollees (mean age ± standard deviation (SD) of 53.4 ± 7.1 years; 57.9% male; 70.3% diagnosed in the outpatient setting; Table 1). Enrollees were followed for a mean of 3.4 ± 0.8 years after the index diagnosis, and 7.6% had a preventable HF hospitalization. During the full observation period, enrollees had a total of 44,296 medication fills, and were prescribed an average of 1.7 ± 0.7 HF-related medication categories. Of the 14,157 (53.5%) enrollees who were prescribed more than one of the medication category during the observation period, the top three medication category combinations prescribed at any point were as follows: ACEI/ARBs and beta blockers (66.0% of enrollees taking multiple medication categories); ACEI/ARBs, beta blockers, and aldosterone receptor antagonists (17.8%); ACEI/ARBs and aldosterone antagonists (5.1%).

**Table 1:** Characteristics of all enrollees by long-term adherence, MarketScan 2008–2012 (N = 26,439).

Characteristic	N (%)	Mean PDC (%)	Mean PDC category (for observation years 1–5), n (%)			*P*^*a*^
			Poor adherence (<40%)	Moderate adherence (40- <80%)	Good adherence (≥80%)	
***Enrollee Characteristics (n = 26*,*439)***						
All enrollees	26,439 (100.0)	71.2	4,640 (17.6)	8,830 (33.4)	12,969 (49.0)	
Age						
18–34	558 (2.1)	52.7	215 (38.5)	209 (37.5)	134 (24.0)	<0.0001
35–44	2,477 (9.4)	60.7	722 (29.2)	960 (38.8)	795 (32.2)	
45–54	9,193 (34.8)	69.3	1,828 (19.9)	3,365 (35.7)	4,000 (43.5)	
55–64	14,211 (53.8)	74.9	1,985 (14.0)	4,897 (34.5)	7,329 (51.6)	
Sex						
Male	15,317 (57.9)	72.8	2,495 (16.3)	5,362 (35.0)	7,460 (48.7)	<0.0001
Female	11,122 (42.1)	69.0	2,255 (20.3)	4,069 (36.6)	4,798 (43.1)	
Region						
Northeast	2,607 (9.9)	75.1	392 (15.0)	734 (28.2)	1,481 (56.8)	<0.0001
North central	6,517 (24.6)	73.5	1,025 (15.7)	2,020 (31.0)	3,471 (53.3)	
South	13,084 (49.5)	68.4	2,578 (19.7)	4,755 (36.3)	5,751 (44.0)	
West	4,231 (16.0)	73.7	645 (15.2)	1,313 (31.0)	2,274 (53.7)	
Employment status						
Full-time	15,202 (57.5)	71.3	2,616 (17.2)	5,121 (33.7)	7,465 (49.1)	<0.0001
Part-time	274 (1.0)	69.5	51 (18.6)	100 (36.5)	123 (44.9)	
Disabled	4,061 (15.4)	74.5	616 (18.6)	1,213 (29.9)	2,232 (55.0)	
Retired	888 (3.5)	76.1	116 (13.1)	272 (30.6)	500 (56.3)	
Other	6,014 (22.8)	68.0	1,241 (20.6)	2,124 (35.3)	2,649 (44.1)	
County income						
Low (<$38,952)	3,631 (13.7)	66.1	846 (23.3)	1,363 (37.6)	1,422 (39.2)	<0.0001
Medium ($38,952-<$46,334)	6,823 (25.8)	70.5	1,263 (18.5)	2,468 (36.2)	3,092 (45.3)	
High (≥$46,334)	15,985 (60.5)	72.7	2,637 (16.5)	5,593 (35.0)	7,755 (48.5)	
Index diagnosis setting						
Inpatient	7,866 (29.8)	68.4	1,555 (19.8)	2,842 (36.1)	3,469 (44.1)	<0.0001
Outpatient	18,573 (70.3)	72.4	3,085 (16.6)	5,988 (32.2)	9,500 (51.2)	
Charlson Comorbidity Index						
0	340 (1.3)	59.5	105 (30.9)	117 (34.4)	118 (34.7)	<0.0001
1	8,416 (31.8)	72.2	1,424 (16.9)	2,668 (31.7)	4,324 (51.4)	
2	4,092 (15.5)	70.3	764 (18.7)	1,364 (33.3)	1,964 (48.0)	
3	6,093 (23.1)	72.3	990 (16.3)	2,013 (33.0)	3,090 (50.7)	
4	3,197 (12.1)	71.6	543 (17.0)	1,079 (33.8)	1,575 (49.3)	
5+	4,301 (16.3)	69.0	813 (18.9)	1,587 (36.9)	1,901 (44.2)	
Number of included HF-related medication categories being filled						
1	12,282 (46.5)	71.2	2,508 (20.4)	3,307 (26.9)	6,467 (52.7)	<0.0001
2	10,760 (40.7)	71.6	1,640 (15.2)	4,010 (37.3)	5,110 (47.5)	
3	3,133 (11.9)	70.8	429 (13.7)	1,378 (44.0)	1,326 (42.3)	
4	225 (0.9)	61.3	51 (22.7)	114 (50.7)	60 (26.7)	
5	39 (0.2)	53.6	12 (30.8)	21 (53.9)	6 (15.4)	
***Enrollee Outcome Assessment (n = 26*,*439)***						
Preventable HF hospitalization						
Yes	2,014 (7.6)	68.0	392 (19.5)	778 (38.6)	844 (41.9)	<0.0001
No	24,425 (92.4)	71.4	4,248 (17.4)	8,052 (33.0)	12,125 (49.6)	
***Medication Characteristics (n = 44*,*296 medication preparations)***						
ACEI/ARB	23,748 (53.6)	72.2	3,803 (16.0)	8,121 (34.2)	11,824 (49.8)	<0.0001
Beta blockers	14,914 (33.7)	70.8	2,476 (17.1)	5,459 (36.6)	6,979 (46.8)	
Aldosterone receptor antagonists	4,152 (9.4)	68.6	701 (16.9)	1,754 (42.4)	1,696 (40.9)	
Hydralazine	1,218 (2.7)	62.0	293 (24.1)	557 (45.7)	368 (30.2)	
Isosorbide dinitrate	264 (0.6)	60.7	66 (25.0)	131 (49.6)	67 (25.4)	
***Top medication category combinations among enrollees filling more than one medication (n = 14*,*157)***						
ACEI/ARB and beta blockers	9,344 (66.0)	72.7	1,336 (14.3)	3,389 (36.3)	4,619 (49.4)	
ACEI/ARB, beta blockers, and aldosterone antagonists	2,518 (17.8)	72.3	307 (12.2)	1,080 (42.9)	1,131 (44.9)	
ACEI/ARB and aldosterone antagonists	726 (5.1)	66.6	124 (17.1)	330 (45.5)	272 (37.5)	

Abbreviations: ACEI/ARB, angiotensin-converting enzyme inhibitors/angiotensin-receptor blocker; HF, heart failure; PDC, proportion of days covered

^a^ Statistical significance was tested using the Chi-square test or Fisher’s exact test.

### Medication adherence

Enrollees’ average long-term adherence (observation years 1–5) was 71.2% ± 28.1. Additionally, 17.6%, 33.4%, and 49.0% of enrollees had poor, moderate, and good adherence, respectively (Table 1). Good adherence was more common among older individuals, males, residents of counties with a higher mean income, those who were diagnosed with HF in the outpatient setting, filled prescriptions for fewer HF medication categories, or were identified as not having a preventable HF hospitalization. The long-term mean PDC was highest for ACEI/ARBs (72.2% ± 27.3) and beta blockers (70.8% ± 27.1), but lowest for isosorbide dinitrate (60.7% ± 25.3).

### Long-term adherence and long-term risk of preventable HF hospitalizations

**Fig 3:**
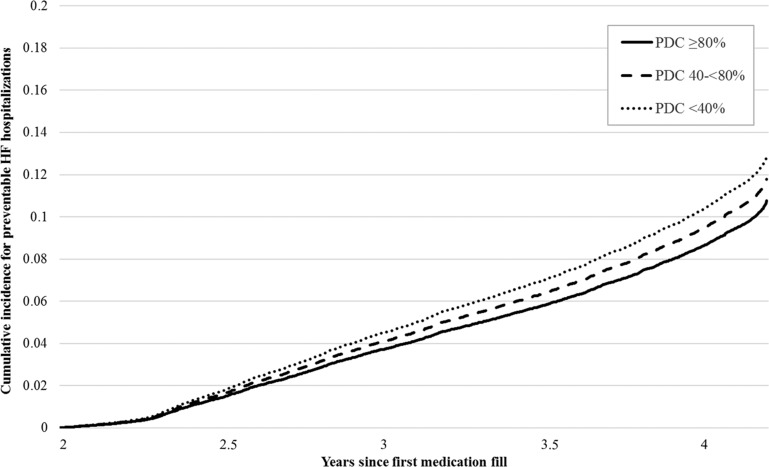
Adjusted cumulative incidence for preventable HF hospitalizations among enrollees at risk for preventable HF hospitalizations, by long-term PDC category, MarketScan 2008–2012 (n = 26,439).

Compared to enrollees with long-term poor adherence, those with long-term good adherence were 15% less likely to have a preventable HF hospitalization during observation years 3–5 (HR 0.85; 95% CI, 0.75–0.96) (Table 2). Those with long-term moderate adherence did not have significantly lower risk compared to individuals with long-term poor adherence (HR 0.94; 95% CI, 0.83–1.07). Individuals who were significantly less likely to have a preventable HF hospitalization included those diagnosed in the outpatient setting (HR 0.42; 95% CI, 0.39–0.47) compared to inpatient setting (Table 3). Additionally, those with a comorbidity score ≥5 compared to a comorbidity score of 0 were significantly more likely to have a preventable HF hospitalization (HR 2.26; 95% CI, 1.24–4.11). Moreover, risk of hospitalization increased progressively for enrollees prescribed more than one HF-related medication category compared to only one category (HR for five medication categories 3.36; 95% CI, 1.79–6.29). Finally, compared to those aged 18–34 years, enrollees 55–64 years were more likely to be hospitalized (HR 1.74; 95% CI, 1.19–2.56). The adjusted cumulative probability for having a preventable HF hospitalization was 11%, 12%, and 13% for those with long-term good, moderate, and poor adherence, respectively (*P*<0.001) (Fig 3).

**Table 2:** Cox proportional hazard models for risk of preventable HF hospitalizations, MarketScan 2008–2012 (N = 26,439).

Model	Poor adherence (PDC <40%) HR (95% CI)	Moderate adherence (PDC 40-<80%) HR (95% CI)	Good adherence (PDC ≥80%) HR (95% CI)	*P*	Formal test of proportional hazards assumption^d^
**Long-term adherence**^a^					
Unadjusted HR	1.00 (REF)	1.07 (0.95–1.21)	0.83 (0.74–0.94)	<0.0001	
Partially adjusted HR^b^	1.00 (REF)	1.00 (0.89–1.13)	0.74 (0.66–0.84)	<0.0001	
Fully adjusted HR^c^	1.00 (REF)	0.94 (0.83–1.07)	0.85 (0.75–0.96)	0.0189	0.6565
**Short-term adherence**^a^					
Unadjusted HR	1.00 (REF)	0.94 (0.82–1.07)	0.80 (0.70–0.91)	0.0003	
Partially adjusted HR^b^	1.00 (REF)	0.88 (0.77–1.00)	0.72 (0.63–0.82)	<0.0001	
Fully adjusted HR^c^	1.00 (REF)	0.82 (0.72–0.94)	0.77 (0.68–0.88)	<0.0001	0.4273

CI, confidence interval; HR, hazard ratio; PDC, proportion of days covered; REF, reference group

^a^ Long-term adherence assessed PDC during the entire study inclusion period (years 1–5). Short-term adherence only assessed PDC during observation years one and two.

^b^Adjusted for age and sex.

^c^Adjusted for age, sex, employment status, region, county income, number of HF-related medication categories, diagnosis setting, and the Charlson Comorbidity Index.

^**d**^ P<0.05 indicates a violation of the proportional hazards assumption for the fully adjusted model, which was tested by creating time-dependent interaction terms.

**Table 3:** Multivariate Cox proportional hazards model: Risk of preventable HF hospitalizations with full adjustment for baseline covariates (N = 26,439).

Characteristic	% with preventable HF hospitalization	HR	95% CI	*P*
Age (years)				
18–34	4.84	1.00 (REF)		
35–44	4.72	0.95	0.62–1.44	0.8042
45–54	6.42	1.26	0.85–1.85	0.2471
55–64	9.01	1.74	1.19–2.56	0.0047
Sex				
Male	7.37	0.98	0.89–1.07	0.5935
Female	7.80	1.00 (REF)		
Region				
Northeast	5.69	1.00 (REF)		
North Central	8.52	1.19	0.99–1.43	0.0660
South	7.34	1.06	0.88–1.26	0.5589
West	8.31	1.24	1.02–1.52	0.0345
Employment status				
Full-time	6.61	1.00 (REF)		
Part-time	7.66	1.08	0.70–1.66	0.7391
Disabled	9.33	1.19	1.05–1.34	0.0080
Retired	11.94	1.39	1.12–1.72	0.0031
Other	8.36	1.32	1.19–1.48	<0.0001
County income				
Low (<$38,952)	7.45	1.06	0.93–1.22	0.3890
Medium ($38,952-<$46,334)	8.21	1.12	1.01–1.24	0.0372
High (≥$46,334)	7.42	1.00 (REF)		
Index diagnosis setting				
Inpatient	13.56	1.00 (REF)		
Outpatient	5.10	0.42	0.39–0.47	<0.0001
Charlson Comorbidity Index				
0	3.24	1.00 (REF)		
1	5.16	1.16	0.64–2.11	0.6316
2	5.50	1.09	0.59–1.99	0.7902
3	7.91	1.60	0.88–2.91	0.1242
4	9.95	1.81	0.99–3.31	0.0541
5+	12.65	2.26	1.24–4.11	0.0076
Number of included HF-related medication categories				
1	4.94	1.00 (REF)		
2	8.47	1.48	1.34–1.64	<0.0001
3	14.20	2.30	2.03–2.60	<0.0001
4	18.22	2.82	2.05–3.87	<0.0001
5	25.64	3.36	1.79–6.29	0.0002
Mean PDC category (long-term adherence)				
Poor adherence (PDC <40%)	8.45	1.00 (REF)		
Moderate adherence (PDC 40-<80%)	8.81	0.94	0.83–1.07	0.3032
Good adherence (PDC ≥80%)	6.51	0.85	0.75–0.96	0.0045

Abbreviations: HF, heart failure; CI, confidence interval; HR, hazards ratio; PDC, proportion of days covered; REF, reference group

Secondary analyses determined the association between long-term medication adherence and long-term risk of preventable HF hospitalizations for the three most frequent medication combinations (S1 Table). HRs were non-significant for these analyses, except those with good adherence to ACEI/ARBs and aldosterone receptor antagonists were 47% less likely to have a preventable HF hospitalization than those with poor adherence (HR 0.53; 95% CI 0.28–1.00).

### Sensitivity analyses

Short-term adherence was assessed during observation years 1 and 2, with enrollees having a mean PDC of 73.2 ± 25.1 and 13.6%, 36.7%, and 49.7% of enrollees being classified as having poor, moderate, and good adherence, respectively (S2 Table). When we examined the relationship between individuals’ adherence categories in the short-term and long-term, we found that among enrollees who were short-term moderate adherers, 15.9% were re-classified as good adherers and 24.2% were re-classified as poor adherers in the long term (Table 4). Additionally, 80.5% of short-term good adherers were also long-term good adherers, and 79.8% of short-term poor adherers were also long-term poor adherers.

**Table 4:** Enrollees’ short-term and long-term adherence category, MarketScan 2008–2012 (N = 26,439).

	Long-term adherence^b^		
	Good n (% of short-term category)	Moderate n (% of short-term category)	Poor n (% of short-term category)
**Short-term good adherence**^**a**^	2,890 (80.5)	578 (16.1)	121 (3.4)
**Short-term moderate adherence**	1,547 (15.9)	5,804 (59.8)	2,350 (24.2)
**Short-term poor adherence**	203 (1.5)	2,448 (18.6)	10,498 (79.8)

^a^Short-term adherence defined as average medication adherence during observation years one and two.

^b^Long-term adherence defined as average medication adherence during observation years 1–5.

Our model of short-term adherence and its association with long-term preventable HF hospitalization risk demonstrated a similar association to the primary model of long-term adherence. Compared to poor adherers, short-term good adherers were 23% less likely to be hospitalized in the long-term compared to short-term poor adherers (HR 0.77; 95% CI 0.68–0.88). Additionally, those with moderate adherence were 18% less likely (HR 0.82; 95% CI 0.72–0.94) (Table 2). Further analyses of the short-term moderate adherence group found that in comparison to individuals who remained moderate adherers in the long-term, those who were re-classified as long-term good adherers were 35% less likely to have a preventable HF hospitalization ((HR 0.65; 95% CI 0.54–0.79); S3 Table).

### Discussion

Medication adherence is an important component of self-care for HF and preventing adverse health outcomes. However, we found that only about half of our cohort with newly diagnosed HF had good long-term adherence (1–5 years) to the five medication categories recommended by the ACC/AHA’s 2013 national guideline for HF management. Individuals with good long-term adherence had significantly decreased risk (15% lower risk) of having a long-term preventable HF hospitalization (during years 3–5) compared to poor adherers. Moreover, this study is the first to demonstrate that having moderate or good short-term adherence (1–2 years) is associated with decreased risk for having a preventable HF hospitalization in the long term, but having moderate long-term adherence is not. Further investigation of the moderate short-term adherence group found that only 60% of individuals in this group were also classified as having moderate long-term adherence, and the remaining 16% and 24% had good or poor long-term adherence, respectively. Persons who initially had moderate short-term adherence and then good long-term adherence had significantly decreased risk of hospitalizations compared to the other groups, including the sustained moderate adherence group (35% lower risk). Therefore, maximizing medication adherence beyond two years is important, and further studies that investigate the types of interventions needed to achieve and sustain adherence among this population are warranted.

Overall, 7% of our cohort had HF hospitalizations designated by federal quality indicators as potentially preventable and certain demographic and clinical subgroups were at higher risk of having these events. Our use of a definition that identifies unplanned, acute HF hospitalizations, is a more specific case definition that better classifies hospitalizations that may have been prevented had medication non-adherence been effectively addressed. Although HF is a chronic and progressive condition and some HF-related hospitalizations are likely unavoidable, many of these events are preventable through the provision of high-quality outpatient care [3]. This highlights an important opportunity for focused attention and intervention. An analysis of a large HF registry found that medication and/or dietary non-adherence (e.g. non-adherence to sodium reduction recommendations) were precipitating causes for 10.3% of all HF hospitalizations [32]. Additionally, it is estimated that the U.S. Centers for Medicare and Medicare Services (CMS) Hospital Readmissions Reduction Program resulted in an overall decline in readmissions for its targeted conditions from 21.5% to 17.8% (3.7% difference) from 2007–2015 [33]. Our findings suggest an even greater potential impact in reducing readmissions through improving medication adherence alone. Although there are potential limitations in using the U.S. CMS Hospital Readmissions Reduction Program measures to quantify HF readmission rates [34], these findings, coupled with our results, support that systematically measuring for and addressing medication non-adherence to avoid or delay progression of HF is essential to reduce the risk for hospitalizations and can supplement existing efforts to decrease HF readmission rates.

This study identified differences in adherence across the five class-I medication categories for HFrEF. ACEI/ARB was the most frequently used medication category (53.6% of all medication preparations). Even though this category had the highest adherence rate (mean PDC = 72.2%), only half (49.8%) of patients who used these types of medications were characterized as having good adherence. The lowest rates of utilization and adherence among the five medication categories where for hydralazine (2.7% of all medication preparations; mean PDC = 62.0%) and isosorbide dinitrate (0.6% of all medication preparations; mean PDC = 60.7%). The low utilization rates may be because class-I guidelines for hydralazine and isosorbide dinitrate use in combination are specifically for African Americans [7, 35], but we were unable to examine utilization by race/ethnicity because the information is not captured in MarketScan data. A previous study found that more than half of HF patients prescribed hydralazine and isosorbide dinitrate at discharge did not fill these prescriptions within 90 days [36]. The poor adherence with these medications may in part be related to multiple doses per day regimen, which is also known to be associated with poor adherence [37]. In addition, poor tolerance or side effects that require dose adjustments to these medications may contribute to poor adherence as measured by PDC [38, 39].

Adherence also varied by demographic characteristics, including being particularly low among the youngest age groups. A prior study found that forgetfulness and being too busy were the most commonly reported reasons for being nonadherent to cardiovascular medications among commercially-insured adults [40]. Strategies, such as incorporating medication taking with routine daily activities, reducing therapeutic complexity, and using smartphone medication reminder applications and mail order pharmacies can be used to help overcome these particular barriers among younger adults [37, 40, 41]. Furthermore, the majority of the enrollees assessed in this study were fully employed, of whom only half had good adherence. Employers may consider providing integrated healthcare and pharmacy services within the workplace, which has been shown to support adherence to chronic disease medications [42–44]. Our study also found that enrollees who were taking multiple medication categories had worse adherence and were more likely to have a preventable HF hospitalization. Individuals who are taking multiple medication categories may also have a more severe condition, which emphasizes the importance of increased adherence to medications. Prescribing strategies such as fixed-dose combinations [37], as well as pharmacist-led medication reconciliation and counseling can help overcome some of the medication burden barriers that HF patients face in order to improve adherence [37, 45].

This study has several limitations. First, MarketScan Commercial data is a convenience sample of select employer and commercial health plans, and is not necessarily generalizable to all individuals with private health insurance plans or those with public health insurance plans (i.e., Medicaid). Second, MarketScan Commercial data does not contain data to assess ejection fraction, so we were unable to limit analyses to those with HFrEF. However, HF with preserved ejection fraction is less common among younger adults [46], we adjusted for comorbidity burden and the index diagnosis setting, which likely serve as proxies for disease severity, and medication adherence has been shown to be a predictor of HF hospitalizations independent of ejection fraction [47]. Additionally, HF hospitalization rates have been found to be high among HF patients with preserved and reduced ejection fraction, which emphasizes the need for using effective strategies to improve medication adherence among all HF patients [8]. Other limitations of the data source include the lack of data on other potential confounders, such as race/ethnicity and indicators of patient-reported health factors (e.g., health related quality of life), as well as lack of data on deaths that occurred outside of the hospital. Third, an overall limitation of all prescription claims data when measuring medication adherence is that it captures the act of filling prescriptions but does not necessarily reflect patients taking their medications as prescribed. Furthermore, it does not provide data on reasons why an individual was non-adherent. Thus, PDC could not account for nonadherence that was due to medication intolerance. A prior study of HF patients estimated that 45% of medication discontinuation was due to clinical factors and/or patient-reported adverse reactions [48]. Fourth, we applied commonly used cutpoints for defining medication adherence in claims data, but future research should determine evidence-based PDC cutpoints specifically for heart failure patients’ health outcomes. Fifth, we excluded enrollees with HF hospitalizations within two years of their diagnosis to assess long-term risk and construct a more uniform cohort, but this likely establishes a lower-risk sample. Our study’s findings could be different if applied to the excluded enrollees, because there may be other disease conditions or behaviors that influence their hospitalizations within two years of their diagnosis. Sixth, we did not control for medication costs in our models; prescription co-pays may have an effect on adherence for HF patients [49, 50]. Finally, national federal efforts and overall increased attention of hospitals to decrease re-hospitalization rates for HF patients during our study period may have indirectly influenced the hospitalizations rates that we observed [33]. However, our results indicate that there are still important opportunities to improve medication adherence among HF patients, which can further support these efforts to decrease hospitalization rates.

In conclusion, this study found that roughly half of individuals aged 18–64 years enrolled in commercial and employer-based health insurance plans with newly diagnosed HF and prescribed at least one guideline-recommended HF medication category had good adherence to their medication. Compared to enrollees with poor long-term adherence to these medications, those with good adherence had 15% lower risk of having a potentially preventable HF hospitalization. Future studies are needed to identify evidence-based interventions that support sustained medication adherence among this population. Use of these types of interventions are likely key in effectively managing this high-cost chronic condition and improving health outcomes.

## Supporting information

S1 Table Cox proportional hazard models for the association between long-term adherence (observation years 1–5) and long-term risk of preventable HF hospitalizations (observation years 3–5), by the most common medication combinations, MarketScan 2008–2012. (DOCX)

S2 Table Characteristics of all enrollees by short-term adherence, MarketScan 2008–2012 (N = 26,439). (DOCX)

S3 Table Cox proportional hazard models for long-term risk of preventable HF hospitalizations, for short-term moderate adherers only, MarketScan 2008–2012 (n = 9,701). (DOCX)
