# Supplementary material for: Association between long-term adherence to class-I recommended medications and risk for potentially preventable heart failure hospitalizations among younger adults
Source: PLoS One. 2019 Sep 23;14(9):e0222868. doi: 10.1371/journal.pone.0222868 (PMC6756532; doi:10.1371/journal.pone.0222868)
Supplement: S1 Table — (DOCX) [file pone.0222868.s001.docx]

**S1 Table.** **Cox proportional hazard models for the association between long-term adherence (observation years 1-5) and long-term risk of preventable HF hospitalizations (observation years 3-5), by the most common medication combinations, MarketScan 2008-2012**

| **Model** | **Poor adherence (PDC <40%)**  **HR (95% CI)** | **Moderate adherence (PDC 40-<80%)**  **HR (95% CI)** | **Good adherence (PDC ≥80%)**  **HR (95% CI)** | ***P*** | **Formal test of proportional hazards assumption^‡^** |
| --- | --- | --- | --- | --- | --- |
| **ACEI/ARB and beta blockers (n=10,158)** | | | | | |
| Unadjusted HR | 1.00 (REF) | 1.09 (0.88-1.36) | 0.96 (0.77-1.18) | 0.2563 |  |
| Partially adjusted HR* | 1.00 (REF) | 1.03 (0.83-1.28) | 0.87 (0.70-1.09) | 0.1117 |  |
| Fully adjusted HR^†^ | 1.00 (REF) | 1.07 (0.86-1.34) | 0.98 (0.79-1.22) | 0.5154 | 0.2730 |
| **ACEI/ARB, beta blockers, and aldosterone receptor antagonists (n=2,777)** | | | | | |
| Unadjusted HR | 1.00 (REF) | 0.77 (0.57-1.04) | 0.83 (0.61-1.12) | 0.2418 |  |
| Partially adjusted HR* | 1.00 (REF) | 0.72 (0.53-0.98) | 0.76 (0.56-1.03) | 0.1087 |  |
| Fully adjusted HR^†^ | 1.00 (REF) | 0.72 (0.53-0.98) | 0.79 (0.58-1.08) | 0.1120 | 0.5799 |
| **ACEI/ARB and aldosterone receptor antagonists (n=799)** | | | | | |
| Unadjusted HR | 1.00 (REF) | 0.85 (0.49-1.48) | 0.61 (0.33-1.12) | 0.2456 |  |
| Partially adjusted HR* | 1.00 (REF) | 0.70 (0.40-1.23) | 0.52 (0.28-0.96) | 0.1117 |  |
| Fully adjusted HR^†^ | 1.00 (REF) | 0.63 (0.35-1.15) | 0.53 (0.28-1.00) | 0.1381 | 0.2663 |

Abbreviations: ACEI/ARB = angiotensin-converting enzyme inhibitors/angiotensin-receptor blockers; CI = confidence interval; HR = hazards ratio; PDC = proportion of days covered; REF = reference group

*Adjusted for age and sex.

† Adjusted for age, sex, employment status, region, county income, number of HF-related medication categories (if applicable), diagnosis setting, Charlson Comorbidity Index.

**‡** P<0.05 indicates a violation of the proportional hazards assumption.
