# Supplementary material for: Association between long-term adherence to class-I recommended medications and risk for potentially preventable heart failure hospitalizations among younger adults
Source: PLoS One. 2019 Sep 23;14(9):e0222868. doi: 10.1371/journal.pone.0222868 (PMC6756532; doi:10.1371/journal.pone.0222868)
Supplement: S2 Table — (DOCX) [file pone.0222868.s002.docx]

**S2 Table. Characteristics of all enrollees by short-term adherence, MarketScan 2008-2012 (N=26,439)**

| **Characteristic** | **N (%)** | **Mean PDC (%)** | **Mean PDC category (observation years 1-2), n (%)** | | | ***P*** |
| --- | --- | --- | --- | --- | --- | --- |
|  |  |  | **Poor**  **adherence**  **(<40%)** | **Moderate adherence**  **(40- <80%)** | **Good adherence (≥80%)** |  |
| ***Enrollee Characteristics (n=26,439)*** | | | | | | |
| All enrollees | 26,439 (100.0) | 73.2 | 3,589 (13.6) | 9,701 (36.7) | 13,149 (49.7) |  |
| Age | | | | | | |
| 18-34 | 558 (2.1) | 59.0 | 174 (31.2) | 221 (39.6) | 163 (29.2) | <0.0001 |
| 35-44 | 2,477 (9.4) | 65.2 | 556 (22.5) | 984 (39.7) | 937 (37.8) |  |
| 45-54 | 9,193 (34.8) | 71.8 | 1,364 (14.8) | 3,496 (38.0) | 4,333 (47.1) |  |
| 55-64 | 14,211 (53.8) | 76.1 | 1,495 (10.5) | 5,000 (35.2) | 7,716 (54.3) |  |
| Sex | | | | | | |
| Male | 15,317 (57.9) | 74.4 | 1,879 (12.3) | 5,572 (36.4) | 7,866 (51.4) | <0.0001 |
| Female | 11,122 (42.1) | 71.6 | 1,710 (15.4) | 4,129 (37.1) | 5,283 (47.5) |  |
| Region | | | | | | |
| Northeast | 2,607 (9.9) | 75.5 | 313 (12.0) | 865 (33.2) | 1,429 (54.8) | <0.0001 |
| North Central | 6,517 (24.6) | 75.1 | 778 (11.9) | 2,277 (35.0) | 3,462 (53.1) |  |
| South | 13,084 (49.5) | 71.3 | 1,989 (15.2) | 5,060 (38.7) | 6,035 (46.1) |  |
| West | 4,231 (16.0) | 74.9 | 507 (12.0) | 1,499 (35.5) | 2,225 (52.6) |  |
| Employment status | | | | | | |
| Full-time | 15,202 (57.5) | 73.1 | 2,014 (13.4) | 5,654 (37.2) | 7,507 (49.4) | <0.0001 |
| Part-time | 274 (1.0) | 72.0 | 41 (15.0) | 106 (38.7) | 127 (46.4) |  |
| Disabled | 4,061 (15.4) | 75.8 | 474 (11.7) | 1,341 (33.0) | 2,246 (55.3) |  |
| Retired | 888 (3.5) | 77.5 | 87 (9.8) | 302 (34.0) | 499 (56.2) |  |
| Other | 6,014 (22.8) | 71.1 | 946 (15.7) | 2,298 (38.2) | 2,770 (46.1) |  |
| County income | | | | | | |
| Low (<$38,952) | 3,631 (13.7) | 69.6 | 606 (16.7) | 1,446 (39.8) | 1,579 (43.5) | <0.0001 |
| Medium ($38,952-<$46,334) | 6,823 (25.8) | 72.6 | 966 (14.2) | 2,540 (37.3) | 3,317 (48.6) |  |
| High (≥$46,334) | 15,985 (60.5) | 74.3 | 2,009 (12.6) | 5,716 (35.8) | 8,260 (51.7) |  |
| Index diagnosis setting | | | | | | |
| Inpatient | 7,866 (29.8) | 70.7 | 1,186 (15.1) | 3,144 (40.0) | 3,536 (45.0) | <0.0001 |
| Outpatient | 18,573 (70.3) | 74.3 | 2,403 (12.9) | 6,557 (35.3) | 9,613 (51.8) |  |
| Charlson Comorbidity Index | | | | | | |
| 0 | 340 (1.3) | 73.3 | 94 (27.7) | 92 (27.1) | 154 (45.3) | <0.0001 |
| 1 | 8,416 (31.8) | 73.3 | 1,144 (13.6) | 3,009 (35.8) | 4,263 (50.7) |  |
| 2 | 4,092 (15.5) | 72.7 | 592 (14.5) | 1,477 (36.1) | 2,023 (49.4) |  |
| 3 | 6,093 (23.1) | 73.8 | 758 (12.4) | 2,272 (37.3) | 3,063 (50.3) |  |
| 4 | 3,197 (12.1) | 73.6 | 420 (13.1) | 1,174 (36.7) | 1,603 (50.1) |  |
| 5+ | 4,301 (16.3) | 72.2 | 581 (13.5) | 1,675 (39.0) | 2,045 (47.5) |  |
| Number of included HF-related medication categories being filled | | | | | | |
| 1 | 12,282 (46.5) | 73.2 | 2,045 (16.7) | 3,830 (31.2) | 6,407 (52.2) | <0.0001 |
| 2 | 10,760 (40.7) | 73.5 | 1,210 (11.3) | 4,306 (40.0) | 5,244 (48.7) |  |
| 3 | 3,133 (11.9) | 72.8 | 297 (9.5) | 1,419 (45.3) | 1,417 (45.2) |  |
| 4 | 225 (0.9) | 66.2 | 32 (14.2) | 123 (54.7) | 70 (31.1) |  |
| 5 | 39 (0.2) | 62.3 | 5 (12.8) | 23 (59.0) | 11 (28.2) |  |
| ***Enrollee Outcome Assessment (n=26,439)*** | | | | | | |
| Preventable HF hospitalization | | | | | | |
| Yes | 2,014 (7.6) | 70.2 | 313 (15.5) | 812 (40.3) | 889 (44.1) | <0.0001 |
| No | 24,425 (92.4) | 73.4 | 3,276 (13.4) | 8,889 (36.4) | 12,260 (50.2) |  |
| ***Medication Characteristics (n=44,296 medication preparations)*** | | | | | | |
| ACEI/ARB | 23,748 (53.6) | 73.9 | 2,940 (12.4) | 8,846 (37.3) | 11,92 (50.4) | <0.0001 |
| Beta blockers | 14,914 (33.7) | 72.9 | 1,834 (12.3) | 5,908 (39.6) | 7,172 (48.1) |  |
| Aldosterone receptor antagonists | 4,152 (9.4) | 71.3 | 500 (12.0) | 1,810 (43.6) | 1,842 (44.4) |  |
| Hydralazine | 1,218 (2.7) | 66.5 | 200 (16.4) | 600 (49.3) | 418 (34.3) |  |
| Isosorbide dinitrate | 264 (0.6) | 66.3 | 35 (13.3) | 142 (53.8) | 87 (33.0) |  |
| ***Top medication category combinations among enrollees filling >1 medication (n=14,157)*** | | | | | | |
| ACEI/ARB and beta blockers | 9,344 (66.0) | 74.2 | 998 (10.7) | 3,669 (39.3) | 4,677 (50.1) |  |
| ACEI/ARB, beta blockers, and aldosterone antagonists | 2,518 (17.8) | 73.8 | 220 (8.7) | 1,097 (43.6) | 1,201 (47.7) |  |
| ACEI/ARB and aldosterone antagonists | 726 (5.1) | 71.2 | 85 (11.7) | 324 (44.6) | 317 (43.7) |  |

Abbreviations: ACEI/ARB = angiotensin-converting enzyme inhibitors/angiotensin-receptor blockers; HF = heart failure; PDC = proportion of days cover
