# Supplementary material for: Association between long-term adherence to class-I recommended medications and risk for potentially preventable heart failure hospitalizations among younger adults
Source: PLoS One. 2019 Sep 23;14(9):e0222868. doi: 10.1371/journal.pone.0222868 (PMC6756532; doi:10.1371/journal.pone.0222868)
Supplement: S3 Table — (DOCX) [file pone.0222868.s003.docx]

**S3 Table.** **Cox proportional hazard models for long-term risk of preventable HF hospitalizations, for short-term moderate adherers only, MarketScan 2008-2012 (n=9,701)**

| **Model** | **Short-term moderate and long-term moderate adherers**  **HR (95% CI)** | **Short-term moderate and long-term good adherers**  **HR (95% CI)** | ***P*** |
| --- | --- | --- | --- |
| Fully adjusted HR^*^ | 1.00 (REF) | 0.65 (0.54-0.79) | <0.0001 |

Abbreviations: CI = confidence interval; HR = hazards ratio; REF = reference group

* Adjusted for age, sex, employment status, region, county income, number of HF-related medication categories, diagnosis setting, Charlson Comorbidity Index.
